# Personal

**Published:** 1906-10

**Authors:** 


### PERSONAL.

Dr. L. S. McMurtry, of Louisville, and Dr. Charles A. L. Reed, of Cincinnati, paid a visit to Buffalo in August, on their way home after attending the Toronto meeting of the British Medical Association, and were the guests of Dr. William Warren Potter, of Franklin Street. Dr. H. E. Hayd, of 493 Delaware Avenue, entertained at dinner one evening in honor of Dr. McMurtry.

Dr. L. G. Hanley, of Buffalo, surgeon to the Sisters of Charity Hospital, received the degree of doctor of laws at the semicentennial of Niagara University held September 26, 1906 at Niagara Falls.

Dr. F. Park Lewis, of Buffalo, who was appointed by Governor Higgins sometime ago a member of the commission for the registration of the blind in the state of New York, was elected president of the commission at a preliminary meeting held at New York September 29, 1906. His colleagues are W. J. McClusky, of Syracuse, and E. Morford, of Brooklyn. The organisation was completed by the selection of O. H. Burritt, superintendent of the state school for the blind at Batavia, as secretary.

Dr. Alexander Hugh Ferguson, of Chicago, has been decorated by the King of Portugal with the insignia of the commandership in the Order of Christ, in recognition of his contributions to surgical science and art. This is the highest distinction that the King of Portugal confers upon a foreigner.

Dr. Nelson W. Wilson, of Buffalo, associate editor of this Journal, was appointed a member of the Literary Committee of the Association of Military Surgeons of the United States at the recent annual meeting held at Buffalo.

Dr. Ernest Wende, of Buffalo, who has been enjoying a vacation in the Restigouche region of Canada, has returned and resumed his professional work. Dr. Wende has been invited to become a collaborator of the American Journal of Dermatology, vice Charles Warrene Allen, deceased.

Dr. William House, of Portland, Ore., formerly of Buffalo, recently paid a visit to his old home and many friends. Dr. House for some time past has been in medical charge of a large sanitarium for the treatment of mental and nervous diseases at Portland.

